# HIF-2α inhibitors in clear cell renal cell carcinoma: a clinical pharmacy perspective on lipid metabolism, therapeutic management, and resistance strategies

**DOI:** 10.3389/fmed.2025.1735808

**Published:** 2025-12-10

**Authors:** Dongmei Chen, Chunwang Hua

**Affiliations:** Department of Pharmacy, Taizhou Second People’s Hospital Affiliated to Yangzhou University, Taizhou, Jiangsu, China

**Keywords:** HIF-2α inhibitors, lipid metabolism, tumor metabolic reprogramming, clinical translation, clear cell renal cell carcinoma (ccRCC)

## Abstract

Renal cell carcinoma (RCC), and clear cell renal cell carcinoma (ccRCC) in particular, is characterized by perturbed lipid metabolism and constitutive activation of hypoxia-inducible factor-2α (HIF-2α). This mini review specifically focuses on ccRCC, which represents 80% of all RCC cases and is uniquely characterized by VHL loss and HIF-2α activation. As a central transcription factor, HIF-2α not only regulates the growth and metastasis of tumor cells but also alters lipid metabolism by activating multiple signaling pathways, thereby promoting tumor progression. Despite the significant advances in our understanding of RCC pathogenesis, there is an urgent need for new targeted therapies. The advance in the design of selective HIF-2α inhibitors has uncovered a novel therapeutic path for ccRCC by direct inhibition of this central oncogenic driver. This mini review outlines how HIF-2α inhibitors exert antitumor effects through specific molecular mechanisms, particularly how they modulate lipid metabolism and related molecular networks. And, the latest clinical trial information is used to determine the effectiveness, safety, and translational potential of the ccRCC as a precision therapy. By integrating existing mechanistic and clinical evidence, the present article intends to instruct the design of future drugs and the optimization of therapeutic modalities so that it might impact the clinical management of ccRCC in the future.

## 1 Introduction

Clear cell renal cell carcinoma (ccRCC), the most common sub-type of renal cell carcinoma, accounts for approximately 80% of all cases of the disease and poses a clinical challenge due to its characteristic metabolic phenotypes (1). Histologically, one of the most significant features of ccRCC is the massive accumulation of intracellular lipids (2). The phenotype is not merely descriptive, but more importantly an underlying change in lipid metabolism (3). Continuous fat production and increased fatty acid intake are achieved by maintaining energy balance, membrane synthesis, and signal activities, providing positive support for tumor progression, invasiveness, and treatment resistance (4). Worldwide, ccRCC has become a significant public health challenge, with a reported incidence of over 400,000 new cases yearly, and its associated mortality rate to be approximately 175,000 yearly (1). Especially for patients with metastasis, the overall survival rate is poor (5). Although recent therapeutic developments, the 5-years survival remains 12%–15%. First-line treatments (tyrosine kinase inhibitors, immune checkpoint inhibitors) lead to objective response rates of 40%–60%, but the response is either very limited (10% complete responses) or the median progression-free survival is seldom exceeding 15 months (6). This indicates the importance of finding novel therapies targeting directly metabolic vulnerabilities that are intrinsic to ccRCC (3).

This unconventional metabolic state is strongly associated with recurrent inactivation of the von Hippel–Lindau (VHL) tumor suppressor gene, which is observed in 90% of advanced ccRCC cases (7). Loss of VHL suppresses proteasomal degradation of the hypoxia-inducible factors (HIFs) and causes their constitutive stabilization, notably of the HIF-2α isoform (7). HIF-2α is the key oncogenic activator by activating angiogenesis-promoting, cell-proliferation-promoting and metabolic re-programming programmes via transcription (8). The VHL–HIF-2α axis drives ccRCC pathogenesis (8). Compared to HIF-1α, which plays a tumor-suppressing role in certain cases, HIF-2α is more prone to oncogenic effects, particularly in the regulation of lipid metabolism (2). Given this, HIF-2α has emerged as an attractive candidate for drug targets, due to it being a valuable source of novel pharmacological targeting approaches (8). Several approaches toward inhibition of HIF-2α have been investigated, including therapeutic ones, such as belzutifan (Welireg), a small-molecule inhibitor that suppresses binding between HIF-2α and ARNT, thus preventing necessary transcriptional activation for angiogenic and tumor proliferation (9). Belzutifan has been shown to be clinically efficacious in both VHL-associated and sporadic ccRCC (9), providing much-needed addition to molecularly targeted treatment options.

**FIGURE 1:**
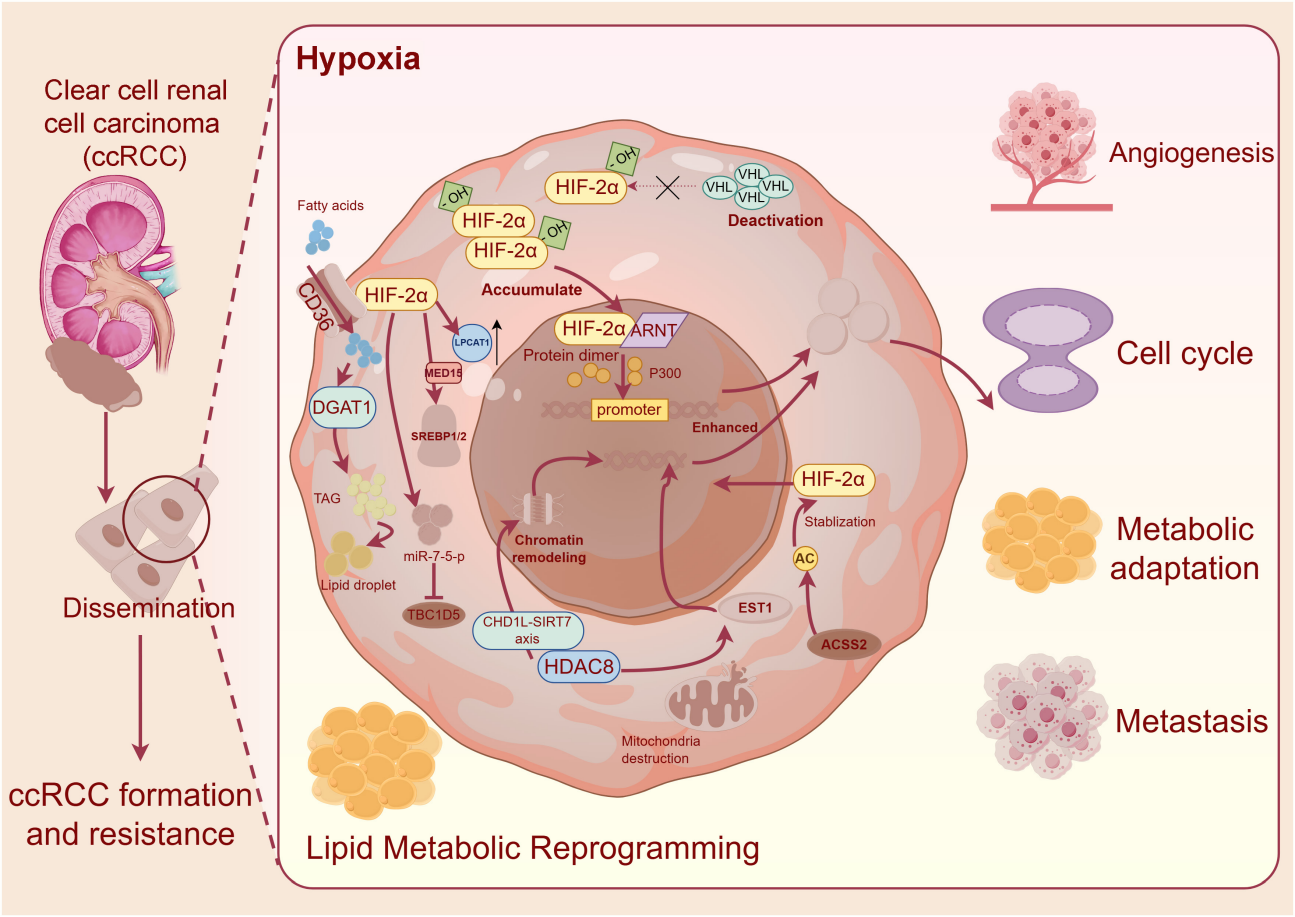
Hypoxia-inducible factor 2α (HIF-2α)–mediated lipid metabolic reprogramming and oncogenic signaling in clear cell renal cell carcinoma (ccRCC). Loss of VHL leads to HIF-2α stabilization, nuclear accumulation, and ARNT dimerization. HIF-2α transcriptionally activates CD36, DGAT1, LPCAT1, and MED15–SREBP1/2 pathways, promoting lipid uptake, synthesis, and storage. Lipophagy is suppressed via miR-7-5p–TBC1D5 inhibition. HDAC8 and CHD1L–SIRT7 enhance HIF-2α transcriptional output. These processes drive angiogenesis, metabolic adaptation, cell cycle progression, metastasis, and therapeutic resistance in ccRCC. VHL, von Hippel–Lindau; HIF-2α, hypoxia-inducible factor 2α; ARNT, aryl hydrocarbon receptor nuclear translocator; CD36, cluster of differentiation 36; DGAT1, diacylglycerol O-acyltransferase 1; LPCAT1, lysophosphatidylcholine acyltransferase 1; MED15, mediator complex subunit 15; SREBP1/2, sterol regulatory element-binding proteins 1 and 2; LD, lipid droplet; TBC1D5, TBC1 domain family member 5; miR-7-5p, microRNA-7-5p; HDAC8, histone deacetylase 8; CHD1L, chromodomain helicase DNA-binding protein 1-like; SIRT7, sirtuin 7.

While HIF-2α dysregulation occurs in various RCC subtypes, this review specifically focuses on ccRCC due to its unique dependency on the VHL-HIF-2α signaling axis, which is present in approximately 90% of cases (10, 11). The metabolic reprogramming and therapeutic targeting strategies discussed herein are primarily relevant to ccRCC, though some principles may extend to other VHL-deficient RCC subtypes (12). In this review, the mechanistic framework for HIF-2α inhibition in ccRCC and with special reference to lipid metabolic process has been discussed, linking with the recent clinical data to show the translational potential and to provide direction for the future development path of treatment targeting HIF-2α (Figure 1).

## 2 HIF-2α in clear cell renal cell carcinoma: from molecular mechanisms to metabolic regulation

### 2.1 The VHL-HIF-2α axis: core pathogenic mechanism in ccRCC

The von Hippel–Lindau (VHL) tumor-suppressor gene, which is mutated in nearly 90% of advanced ccRCC cases, encodes a critical component of an E3-ubiquitin-ligase complex responsible for the oxygen-dependent degradation of HIF-α subunits (13). Under normoxic conditions, prolyl-hydroxylase-domain (PHD) enzymes hydroxylate HIF-α, facilitating its recognition by the VHL complex and subsequent proteasomal degradation (14). When VHL is inactivated, this degradation process is disrupted, resulting in constitutive stabilization and activation of HIF-2α even under normal oxygen tension (13).

In contrast to HIF-1α, which may exhibit context-dependent tumor-suppressive effects, HIF-2α functions as a principal oncogenic driver in ccRCC (8, 15). The tumor-suppressive roles of HIF-1α in clear cell renal cell carcinoma (ccRCC) are mediated by the initiation of pro-apoptotic genes (BNIP3, BNIP3L) and inhibition of c-Myc function. This is in striking contrast to HIF-2α, which, as previously mentioned, promotes c-Myc-driven proliferation (16). Furthermore, HIF-1α is often silenced through either promoter methylation or a loss of chromosome 14q in ccRCC, while HIF-2α expression is often retained (17). Importantly, HIF-2α selectively drives oncogenic programs that were not conferred by HIF-1α, including prolonged expression of VEGFA, progression through the cell cycle via CCND1, and extensive reprogramming in lipid metabolism (18). Indeed, there is evidence that HIF-2α preferentially activates the transcription of genes associated with lipid metabolism, proliferation, and angiogenesis (2). These divergent functions also help explain the characteristic clear cell histology of ccRCC, including lipid accumulation within cells (3). Mechanistically, stabilized HIF-2α forms a heterodimer with ARNT and recruits transcriptional co-activators such as p300 to hypoxia-response elements (HREs), thereby sustaining oncogenic transcriptional activity even in the presence of adequate oxygen (19). In summary, the VHL-HIF-2α signaling axis is not only the foremost pathogenic mechanism of ccRCC, and an important molecular basis for the rationale to develop targeted therapies (8).

### 2.2 HIF-2α-mediated transcriptional networks in lipid metabolism

Hypoxia-inducible factor-2α programs comprehensive lipid-metabolic reprogramming in ccRCC via direct and indirect transcriptional regulation. In the direct action, it can induce the expression of several key genes such as LPCAT1 (phospholipid remodeling), CD36 (fatty-acid uptake) and APOL1 (lipid transport), which eventually increase lipid storage in tumors (2). At the indirect level, HIF-2α upregulates MED15, which is a co-activator that stimulates sterol-regulatory-element-binding-protein (SREBP)-driven fatty-acid production. The interplay between this functional interaction represents a feed-forward regulatory circuit reinforcing *de novo* lipogenesis and contributes to persistent tumor growth (19).

There is also emerging evidence indicating that nuclear speckles, specialized RNA-processing bodies, can physically interact with HIF-2α target genes. These interactions appear to affect transcriptional-output profiles that are prognostic for patient outcomes (20). Outside lipid metabolism, HIF-2α induces VEGFA, CCND1 and CXCR4, all of which stimulate angiogenesis, cell-cycle progression and metastatic dissemination. Additionally, it also plays a role in immune evasion through the regulation of CD8? T-cell infiltration (3, 14). Pharmacologically, belzutifan, a small-molecule inhibitor, acts on the PAS-B domain of HIF-2α to prohibit dimerization with ARNT, thereby inhibiting HIF-2α-mediated transcriptional programs and making it an actionable target, at least in ccRCC (9).

## 3 Molecular mechanisms of HIF-2α in lipid metabolic reprogramming

### 3.1 Lipid uptake and storage regulation

Up-regulation of CD36, a scavenger receptor and fatty-acid transporter, constitutes a key mechanism through which HIF-2α enhances fatty-acid uptake in ccRCC. Elevated CD36 expression is closely associated with hypoxic signaling and HIF-2α activation in tumor tissues, promoting lipid-droplet accumulation through DGAT1-dependent triglyceride synthesis (21). Silencing CD36 reduces lipid deposition and attenuates HIF-2α–driven oncogenic effects, identifying CD36 as a critical mediator of lipid-metabolic remodeling (21).

Lipid droplets (LDs) act as dynamic organelles that protect tumor cells from lipotoxic stress while supporting metabolic flexibility and sustained proliferation. These structures store neutral lipids such as triglycerides and cholesterol esters, thereby contributing to the characteristic clear-cell morphology of ccRCC (22). Furthermore, various regulatory factors including sterol regulatory element-binding protein 1 (SREBP1) and histone methyltransferase SETD8, also further promote *de novo* lipogenesis. Specifically, E2F1-mediated activation of SREBP1, stabilized by USP17-dependent SETD8 activity, enhances LD biogenesis (23, 24). G-protein-coupled receptors such as CMKLR1 also participate in lipid uptake and triglyceride synthesis, and pharmacologic inhibition of CMKLR1 has been shown to reduce lipid storage and suppress tumor growth (25). Collectively, these mechanisms integrate lipid uptake, synthesis and storage under the control of the HIF-2α–CD36 axis, representing a central metabolic vulnerability and a promising therapeutic target in ccRCC (26).

### 3.2 Activation of lipid synthesis pathways

Hypoxia-inducible factor-2α binds to the LPCAT1 promoter region to enhance transcription of this enzyme that catalyzes phosphatidylcholine formation, which is necessary for triglyceride synthesis (2). When LPCAT1 was knocked down, triglyceride production was significantly inhibited, suggesting its role in lipid accumulation in ccRCC cells (2). The regulatory feedback system is one in which induction of the NF-κB target FBXW7 promotes degradation of ATP-citrate lyase (ACLY), thereby modulating the availability of acetyl-CoA for fatty-acid production (2).

Hypoxia-inducible factor-2α is a transcription factor that controls the oxygen level in tissues and can influence lipid accumulation (19). SREBP is a transcriptional co-activator that uses MED15, and it is the principal regulator of cholesterol and fatty acid biosynthesis. MED15 promotes lipid-droplet production and tumor growth via direct SREBP interactions. Specifically, MED15 increase lipogenic enzymes activity e.g., FASN. Additionally, high SREBP activity is maintained by a positive feedback loop induced by MED15 via PLK1 and AKT signaling. The clinical overexpression of MED15 was shown to possess a favorable prognosis in clear cell renal cell carcinoma. This confirms the important role of the HIF-2α–MED15–SREBP axis as a core lipid metabolism reprogramming pathway, thereby providing a basis for it to become a therapeutic target (19).

### 3.3 Regulation of autophagy and lipid turnover

Hypoxia-inducible factor-2α signaling is involved in autophagy and lipid turnover. HIF-2α heightens the expression of hsa-miR-7-5p to suppress TBC1D5, being a tumor suppressor (27). The loss of TBC1D5, which commonly occurs in ccRCC, inhibits autophagic flux and lipophagy, preventing the breakdown of lipid droplets and causing the significant lipid accumulation that is characteristic of the clear cell type (27). On the contrary, TBC1D5 restoration enhances lipophagy, decreases lipid loading, and inhibits cancer cell proliferation and metastasis (27). This suggests that in ccRCC, lipophagy functions as a tumor-suppressive mechanism that is actively inhibited by the HIF-2α/miR-7-5p axis to maintain the lipid-rich phenotype required for tumor progression. Lipophagy plays a dual role in ccRCC, highlighting metabolic vulnerability (26). Basal lipoplasty allows tumors to survive stress whereas the hyperlipoplasty caused by TBC1D5 causes depletion of lipids needed for membrane-making signaling (28). Blocking HIF-2α reverses this imbalance by decreasing miR-7-5p, potentially allowing the TBC1D5-supported lipoblasty to recover (29). The above may cause lipid shortage during early treatment with belzutifan, though resistant cells may thereafter inhibit lipoplasty via alternative mechanisms.

Besides the CD36–DGAT1 axis, HIF-2α directly induces LPCAT1 for phospholipid remodeling and triglyceride biosynthesis (2). Further along, the NF-κB–FBXW7 pathway facilitates the ubiquitination and breakdown of ACLY, creating a feedback mechanism that inhibits overproduction of fatty acids and manages metabolic homeostasis (2). HIF-2α activates MED15, which acts with SREBP1 and SREBP2 to further enhance lipid dependency in tumor cells. It is notable that both SREBP1 and SREBP2 are associated with adverse clinical outcomes (19).

According to a study, HIF-2a/LINC02609/APOL1 axis promotes the addition of lipidic droplets, which protects endoplasmic reticulum integrity, and gives rise to enhanced invasive capacity (30). On the contrary, the inhibition of TBC1D5-dependent lipophagy could release free fatty acids from the lipid droplets into β-oxidation and the TBC1D5 lipophagy modulators in ccRCCs enhances the fatty-rich clear-cell phenotype (27). Overall, the processes of lipid intake, synthesis, storage and turnover collectively form a complete regulatory circuit, which shapes the unique metabolic characteristics of ccRCC. And it affords several opportunities for therapy to exploit the metabolic vulnerabilities induced by HIF-2α activation.

## 4 Mechanism of action of HIF-2α inhibitors and advances in drug development

### 4.1 Molecular targeting and mechanism of HIF-2α inhibition

Hypoxia-inducible factor-2α inhibitors are thought to work by disrupting the essential interaction between the HIF-2α dimerization partner ARNT and HIF-2α necessary for transcriptional activation (31). The US has approved belurafenib, which is the first HIF-2α inhibitor (32). HIF-2α’s PAS-B domain, which contains a large cavity within its hydrophobic core, binds to small molecule inhibitors that allosterically disrupt HIF-2α dimerization with ARNT (33). This conformational change prevents the formation of heterodimers, thereby inhibiting the subsequent transcriptional activation process (34). Belzutifan’s targeted mode of action sets it apart from previous agents that indirectly target upstream regulators or inhibit vascular endothelial growth factor (VEGF) signaling (11).

Drugs from the next generation like casdatifan (AB521), use a similar tactic of allosteric inhibition but have better pharmacokinetics and pharmacodynamics (35). At present, clinical trials are underway where these compounds are being used in combination with tyrosine kinase inhibitors (TKIs) or immune checkpoint inhibitors (ICIs) to enhance anti-tumor efficacy (36). These advances can possibly address resistance mechanisms and reduce off-target toxicities associated with previous therapies and represent an important expansion of the therapeutic landscape (37).

### 4.2 Effects on tumor metabolism and therapeutic resistance

Suppressing HIF-2α much affects tumor metabolism by reducing lipid accumulation and reversing metabolic reprogramming (38). The inhibition of HIF-2α lowers the expression of genes involved in lipid biosynthesis. Though, restoration of the TBC1D5 tumor suppressor reverses HIF-2α–mediated lipid changes (27). Besides lipid metabolism, HIF-2α inhibition disturbs the glycolytic and other anabolic pathways critical for tumor adaptation to hypoxia, causing downregulation of VEGFA, CCND1, and SLC2A1 (39).

In other tumors, there is also a lot of evidence indicating that the inhibition of HIF-2α has extensive metabolic effects. For example, in hepatocellular carcinoma, HIF-2α blockade dampens c-MYC expression and activates apoptotic pathways (40). While in breast cancer, HIF-2α inhibition dampens hypoxia-induced stemness and dampens chemoresistance (41). For example, HIF-2α inhibitors may induce resistance via compensatory pathways and epigenetic remodeling. As an example, HDAC8-mediated deacetylation of ETS1 enhances HIF-2α transcriptional function and drives TKI resistance (42). Additionally, the chromatin remodeler CHD1L increases HIF-2α–driven transcription and its inhibition sensitizes tumors to treatment (43). Research on combined treatment strategies is continuously advancing to address this issue of drug resistance. For instance, dual inhibition of sphingosine-1-phosphate signaling and HIF-2α has proven effective in resistant models (44). Moreover, the combination of HIF-2α inhibitors and ICIs could relieve the hypoxia-induced immunosuppression and reinvigorate antitumor immune responses (45).

Ongoing clinical trials are evaluating belzutifan in combination with TKIs, CDK4/6 inhibitors, and ICIs across multiple treatment settings (36). While monotherapy has yielded limited responses in certain contexts, rationally designed combination regimens hold promise for enhancing therapeutic benefit and overcoming acquired resistance (46).

Significantly, lipid metabolism may be restored via compensatory pathways within cells that have become resistant to HIF-2α inhibitors (39). Initially, belzutifan blocks CD36 and LPCAT1-mediated lipid accumulation, however, the resistant cells do appear capable of reactivating lipogenesis in ways that are HIF-2α-independent. For example, HIF-2α can be made stable by ACSS2, and SREBP1/2 can be directly activated by mTOR signaling to promote lipogenesis in the absence of HIF-2α (26). This compensatory metabolic adaptation enables the tumor cells to retain their hallmark clear cell phenotype despite HIF-2α blockade, raising the question whether other lipid metabolic pathways should be targeted to overcome resistance (47).

### 4.3 From metabolic insights to clinical benefits

Clinical responses to HIF-2α inhibitors correlate with metabolic disruption rather than merely target engagement, as evidenced by the direct relationship between lipid depletion and tumor regression (48). Reductions of the fat metabolism observed in response to belzutifan treatment (inhibition of CD36-dependent uptake of fatty acids and lipid incorporation) and the triglyceride production mediated by LPCAT1 correlate clinically with a radiographic tumor regression and symptomatic recovery (9). This kind of metabolic disruption starves the tumor cells of building blocks for cell membrane and energy production and results in quiescence and cell death (49). Results of multiple clinical trials reflect this relationship between metabolic disruption and therapeutic results (11). A reduction in tumor lipid content has been reported after 4–8 weeks of treatment, with similar reductions in serum free fatty acid and triglyceride levels observed by magnetic resonance spectroscopy (50) In addition, baseline expression of lipid metabolism–associated genes could be able to predict clinical benefit (51). For example, increased LPCAT1 expression was correlated with longer progression free (hazard ratio 0.65, *p* = 0.012), and increased CD36 expression with higher objective response rates (35% vs. 18%, *p* = 0.023) (38). Both of these observations suggest that lipid metabolic biomarkers might be useful for guiding patient choice and measuring response to HIF-2α inhibition (10).

## 5 Clinical trials and application of belzutifan

### 5.1 Clinical efficacy in ccRCC

Belzutifan, an orally administered hypoxia-inducible factor-2α (HIF-2α) inhibitor, represents a significant therapeutic advancement in renal cell carcinoma (9). It received initial approval from the U.S. Food and Drug Administration (FDA) in 2021 for the treatment of von Hippel–Lindau (VHL) disease–associated renal cell carcinoma and was subsequently granted expanded approval in 2023 for advanced sporadic RCC that has progressed after multiple lines of therapy (32). While belzutifan’s FDA approval includes treatment for “advanced RCC,” its mechanism of action specifically targets the VHL-HIF-2α pathway characteristic of ccRCC, and clinical trials have primarily enrolled patients with clear cell histology (52). The relevant clinical trial data are presented in Table 1.

**TABLE 1:** Key clinical trials of HIF-2α inhibitors (belzutifan and next-generation agents) in clear cell renal cell carcinoma (ccRCC).

NCT number	Trial name	Phase	Line of therapy	Sample size (*n*)	Population/ inclusion criteria	Regimen	Comparator/ control arm	mPFS (mo) [HR (95% CI), *p*]	mOS (mo) [HR (95% CI), *p*]	Secondary endpoints (ORR/ DCR/DOR)	Main grade ≥ 3 AEs (%)	References
NCT04195750	LITESPARK-005	III	≥2L	746	Previously treated advanced ccRCC after ≥1 line IO or TKI	Belzutifan 120 mg qd	Everolimus 10 mg qd	5.6 vs. 5.6 (HR 0.75)	–	ORR 22% vs. 3%; DCR 57%; DOR –	Anemia 7%, hypoxia 5%	(53–55)
NCT03634540	LITESPARK-003	II	1L	147	Treatment-naïve advanced ccRCC	Belzutifan + Cabozantinib	None (single-arm)	30.3 (95% CI 16.6–NR)	–	ORR 70% (CR 8%), DOR 28.6 mo	Hypertension 4%, fatigue 3%	(56, 57)
NCT02293980	PT2385	I	≥2L	51	Heavily pretreated ccRCC	Belzutifan monotherapy	None	–	–	ORR 14%, DCR 52%	Anemia 5%, fatigue 4%	(50)
NCT04894617	ARC-20	I	≥2L	120	Previously treated ccRCC	Casdatifan ± Cabozantinib	None	–	–	ORR 33%, DCR 81%	Anemia 6%, hypoxia 4%	(35, 76)
NCT05461652	PEAK-1	III (ongoing)	1L	–	Treatment-naïve ccRCC	Casdatifan + Cabozantinib	Cabozantinib monotherapy	Ongoing	Ongoing	–	–	(77)

AE, adverse event; CR, complete response; DCR, disease control rate; DOR, duration of response; HR, hazard ratio; IO, immune-oncology therapy; mOS, median overall survival; mPFS, median progression-free survival; NR, not reached; ORR, objective response rate; PK/PD, pharmacokinetics/pharmacodynamics; TKI, tyrosine kinase inhibitor.

In the randomized phase 3 LITESPARK-005 trial, belurafenib showed statistically and clinically significant improvements in progression-free survival (PFS) compared to everolimus, with a hazard ratio of approximately 0.75 (53). Although both treatment arms reported a median PFS of 5.6 months at the initial interim analysis, divergence in the Kaplan–Meier survival curves over time and a substantially higher objective response rate (ORR) for belzutifan (approximately 22% vs. 3%–4%) suggested more durable disease control (54). Additionally, the early assessment results of patient-reported outcomes also supported belzutifan, with delayed symptom deterioration and trends indicating improved quality of life (55).

During the second-phase open-label LITESPARK-003 trial, bemzoparib plus cabozantinib used as a first therapy resulted in an objective response rate (ORR) of 70% and a complete response rate of 8% (56). The combination produced a median progression-free survival of 30.3 months (95% CI, 16.6–not reached) and a median duration of response of 28.6 months, and had manageable toxicity (57). These results justify advancing to phase 3 evaluation. Belzutifan had activity against tumors in patients with either germline or somatic VHL mutations indicating the drug is relevant to genetically diverse ccRCC populations (11).

### 5.2 Safety profile and tolerability

Belzutifan exhibits a distinct yet generally favorable safety profile, consistent with its molecular mechanism of action (11). The most frequently reported treatment-related adverse events (TRAEs) include anemia and hypoxia, both considered on-target effects linked to HIF-2α’s role in erythropoiesis and oxygen homeostasis (48). These events are typically manageable with supportive care or dose modifications.

In key clinical trials, the incidence of grade 3–4 adverse events such as hypertension and fatigue was relatively low, and no treatment-related deaths were reported (6). Patient-reported outcomes data from the LITESPARK-005 trial provide additional evidence that belzutifan does not negatively impact patients’ quality of life (QOL) compared with everolimus (55). When belinostat is used in combination with tyrosine kinase inhibitors, it may increase efficacy or toxicity respectively, which necessitates appropriate patient inclusion and targeted management plan in order to attain an improved therapeutic outcome (36).

## 6 Clinical challenges and future directions

### 6.1 Clinical challenges: resistance mechanisms and biomarkers

Though belzutifan has shown benefit therapeutically, clinical implementation of HIF-2α inhibitors suffers from issues with response maintenance and the emergence of intrinsic or acquired resistance (11). Resistance has been shown to be promoted by alternative mechanisms to stabilize HIF-2α independent of the VHL (58). These mechanisms include activation of alternative E3 ubiquitin ligases such as MUL1 and alternative metabolic or signaling adaptations (59). Preclinical work has indicated that if inhibitors of upstream regulatory factors (such as the acetyltransferase ACSS2, which stabilizes HIF-2α through acetylation modification) are combined with drugs that directly target HIF-2α, a better synergistic effect may be achieved (60). Additionally, new tricyclic, more selective HIF-2α inhibitors are being developed to bypass HIF-2α resistance-associated mutations in PAS-B (50).

Epigenetic alterations also contribute to therapeutic resistance (61). For example, HDAC8-mediated deacetylation of ETS1 augments HIF-2α transcriptional activity, while chromatin remodelers such as CHD1L amplify HIF-2α–driven transcriptional programs and may contribute to resistance against tyrosine kinase inhibitors (42, 62). Moreover, HIF-2α activation under hypoxic conditions has been implicated in promoting cellular stemness and chemoresistance through modulation of mitochondrial reactive oxygen species (mtROS) (63, 64). Importantly, these resistance mechanisms often correlate with restoration of lipid accumulation despite continued HIF-2α inhibition (26). For instance, HDAC8-mediated resistance preserves lipid storage through dual mechanisms: enhancing SREBP1 transcriptional activity via direct deacetylation and stabilizing HIF-2α through ETS1 modification (42). In the same manner, the activation of ACSS2 by resistant cells can maintain HIF-2α’s acetylation and stability, restoring fatty acid synthesis (39). This metabolic resilience suggests lipid reprogramming may not just be a consequence, but may indeed be a driver of therapy resistance, suggesting that combined strategies may be necessary to target HIF-2α and dysregulated lipid metabolism (47).

As a consequence of the molecular heterogeneity of this ccRCC with regard to the cellular atmosphere, and expression of HIF-2α itself, reliable biomarker stratification will be needed (65). For instance, increased expression of HIF-2α is correlated with favorable response to therapy while low-expression signifies resistance (66). Additional emerging predictive biomarkers include microRNAs that regulate HIF-2α signaling, including miR-185-5p and miR-223-3p, and long non-coding RNAs (lncRNAs) such as LINC01234 (67). Moreover, it has been proposed that protein biomarkers of HIF-2α downstream effectors, such as CYR61 and SEMA6A would correlate and be informative (68). These proposed biomarkers would need additional ongoing validation in clinical studies to be successfully prospectively target candidate patients to improve precision (69).

### 6.2 Clinical management of resistance

In order to effectively manage the resistance, a clinical program should implement an initial rigorous upfront investigation followed by continual follow-up including genomic testing to confirm VHL status and search for compensatory mechanisms such as HDAC8 and CHD1L, as well as traditional follow-up including clinical and radiologic assessment through RECIST criteria and a more sophisticated approach including serial circulating tumor DNA assays to detect mutations underlying the resistance (70). Based on these results, the treatment plan can be adjusted dynamically. It is recommended that patients with primary resistance, meaning no response to first-line therapy, switch to an alternative therapeutic class early on. It is essential to observe clinical context in acquired resistance cases. We can treat oligoprogression by administering belzutifan and adding local ablative therapy. Combination regimens that target compensatory escape pathways will need to be mechanistically added with systemic progression. Potential treatments include co-therapy with HDAC inhibitor for those having elevated levels of HDAC8, manipulation of S1P signaling, or use of an immune checkpoint inhibitor to counteract the patients immunologic evasion (71).

### 6.3 Future directions: combination strategies and personalized medicine

To overcome the resistance to HIF-2α inhibitors, it is necessary to design a reasonable combination therapy. Pairing HIF-2α inhibition with agents that block compensatory metabolic pathways, such as inhibitors of sphingosine-1-phosphate signaling, which has shown efficacy in preclinical models of drug resistance. Combination strategies involving immune checkpoint inhibitors (ICIs) may also further enhance antitumor immunity by mitigating hypoxia-induced immunosuppression (72). However, the inhibitory effects of such combined strategies on T cell function still need to be carefully evaluated.

Phase III trials are now evaluating belzutifan in combination with tyrosine kinase inhibitors, e.g., cabozantinib and lenvatinib, and also in combination with ICIs in several lines of treatment (73). Early preliminary studies have shown that the combined regimens of belzutifan with TKIs and immunotherapy have demonstrated encouraging efficacy in both newly diagnosed and treated patients (56). Other strategies being tested include bi-targeted agents inhibiting both HIF-2α and c-Myc and new inhibitors of immune checkpoints controlled by HIF-2α (e.g., VISTA) (68, 74). There are active pipelines of new HIF-2α inhibitors in development (37, 75). For instance, in early trials of casdatifan (AB521) the observed objective response rate is around one-third and disease control rate 81% (76). The most common grade 3 or higher adverse events were anemia and hypoxia. The ongoing third phase PEAK-1 study is evaluating whether casasitfin combined with cabozantinib is superior to cabozantinib monotherapy (77).

The Personalized medicine is the frontiers of the next generations in HIF-2α–targeted medicines. Combining whole-genomic sequencing and ctDNA-based circulating biopsy will facilitate precision on-line monitoring and adaptive decision making (70). To further advance the precision medicine, onco-biologists, molecular biologists and computational scientists should cooperate closely together. In the field of renal cell carcinoma, only through the joint efforts of multiple disciplines can the potential of HIF-2α inhibition therapy be more deeply translated into clinical practice (78).

## 7 Conclusion

Hypoxia-inducible factor-2α inhibitors constitute a novel class of targeted therapy for ccRCC. They could play an important role in overcoming existing therapeutic inefficiencies as they can directly inhibit the VHL–HIF-2α oncogenic pathway and downstream lipid metabolic changes. The clinical benefits demonstrated by bevacizumab in monotherapy and the positive results obtained from combination therapy trials have jointly driven the rapid development of this field. Historically, long-duration disease control in refractory populations has been reported with response rates of approximately 22%, although response rates can reach approximately 70% with use in a first line combination. However, the issue of drug resistance still exists. Either intrinsic resistance or acquired resistance may occur due to compensatory signaling pathways for example HDAC8, or epigenetic modifications. Additionally, the specific impact of HIF-2α inhibition on lipid turnover remains to be clarified. Future work will be needed to establish rational combination strategies (both with tyrosine kinase inhibitors and metabolic modulators), as well as validate predictive biomarkers (c.t.DNA and lipidomic signatures). Persistence will be required to continue inter-disciplinary collaborations in order that new mechanistic insights translate to sustained clinical benefit. With upcoming phase III trials and the expected approval of next-generation inhibitors, the future landscape might be set and HIF-2α -inhibition might find its place as another mainstay in the treatment of ccRCC.
